# Structural Relaxation of Li_x_(Ni_0.874_Co_0.090_Al_0.036_)O_2_ after Lithium Extraction down to x = 0.12

**DOI:** 10.3390/ma11081299

**Published:** 2018-07-27

**Authors:** Jian Kang, Shigeomi Takai, Takeshi Yabutsuka, Takeshi Yao

**Affiliations:** 1 Graduate School of Energy Science, Kyoto University, Yoshida-Honmachi, Sakyo-ku, Kyoto 606-8501, Japan; kang.jian.77w@st.kyoto-u.ac.jp (J.K.); stakai@energy.kyoto-u.ac.jp (S.T.); yabutsuka@energy.kyoto-u.ac.jp (T.Y.); 2 Institute of Advanced Energy, Kyoto University, Gokasho, Uji, Kyoto 611-0011, Japan; 3 National Institute of Technology, Kagawa College, Chokushi-cho, Takamatsu, Kagawa 761-8058, Japan

**Keywords:** lithium ion battery, NCA, relaxation analysis

## Abstract

A structural relaxation study has been carried out on Li_x_(Ni_0.874_Co_0.090_Al_0.036_)O_2_ after the electrochemical lithium was extraction down to x = 0.12. These relaxation analyses have been carried out using X-ray diffraction coupled with the Rietveld analysis, assuming two phases (H2 and H3) and co-existence with $R\bar{3}m$ symmetry. The mole fraction of the H3 phase seemed not to vary largely during the relaxation time. As for the lattice constants, both H2 and H3 phases gradually increased the *a*-axis with the relaxation time. On the other hand, H2 and H3 phases increased and decreased the *c*-axis, respectively. The results are compared with that of previously reported Ni-rich NCA.

## 1. Introduction

Lithium ion secondary batteries have high performance such as high energy density, good cycle performance, and are now widely used for the power supply of automobiles or renewable power sources [1,2,3,4]. Li_x_NiO_2_, one of the major cathode materials, has advantages for high energy and power densities as well as cost due to the employment of inexpensive Ni instead of Co, although it has a shortage in cycle performance and thermal stability [5,6,7,8,9]. Ohzuku et al. [10] have reported the phase variation during the charge/discharge processes, i.e., hexagonal phase (H1) for 1 > x > 0.75, monoclinic phase (M) for 0.75 ≥ x ≥ 0.45, hexagonal phase (H2) for 0.45 > x ≥ 0.25, and showed two hexagonal phases (H2 + H3) co-exist in the region 0.25 > x. In this region, relatively larger differences in the *c*-axis between the H2 and H3 phases would restrict the reversible reactions to fail the high density and better cycle performance. On the other hand, NCA (Li(Ni,Co,Al)O_2_; Co and Al doped LiNiO_2_) exhibits good high-temperature stability and cycle performance [11,12,13,14,15,16,17] despite the of co-existence of H2 and H3 phases at the higher potential region as LiNiO_2_ [15]. To achieve larger capacity, it is necessary to investigate the charge/discharge process and structural stability in this higher voltage region. We have previously investigated the structural variability for Li(Ni_0.933_Co_0.031_Al_0.036_)O_2_ (Ni-rich NCA) by means of relaxation analysis using X-ray diffraction accompanied by the Rietveld analysis, revealing that small amounts of cobalt substitution such as 0.031 in the formula improve lithium diffusion in the structure [18].

During the lithium-charged or discharged states, electrode materials possess the kinetically preferred structure, which is sometimes different from the thermodynamically stable structure. We have investigated structural variation of electrode materials after the termination of charging or discharging to evaluate the structural variability. We named this analysis technique “relaxation analysis” and applied it to various electrode materials [18,19,20,21,22,23,24,25,26,27].

Concerning the widely studied composition of NCA has been LiNi_0.8_Co_0.15_Al_0.05_O_2_ to meet safety criteria, an intermediate composition between LiNi_0.8_Co_0.15_Al_0.05_O_2_ and Ni-rich NCA should be studied for actual applications. In the former system, structural change at the highly lithium extracted region has been already investigated [11,15,16,17] and it is known that lithium extraction would lead to decreases in the *c*-length of LiNi_0.8_Co_0.15_Al_0.05_O_2_ [11]. In comparison with LiNi_0.8_Co_0.15_Al_0.05_O_2_, the previously studied one possesses a much larger amount of Ni, which has an advantage in cost due to the smaller amount of expensive Co. In the present study, we employed the composition of NCA as Li(Ni_0.874_Co_0.090_Al_0.036_)O_2_ to study the contribution of cobalt to structural variation during relaxation by comparing with the previous results. In this paper, the lithium extracted composition as x = 0.12 was investigated for Li_x_(NCA)O_2_.

## 2. Materials and Methods

### 2.1. Electrochemical Lithium Extraction

Cathode materials of Li(Ni_0.874_Co_0.090_Al_0.036_)O_2_ have been supplied from Sumitomo Metal Mining Co., Ltd. (Tokyo, Japan). A working electrode was prepared by mixing the cathode material with Acetylene Black (AB) and PVdF power with a weight ratio of 80:10:10, followed by spreading the mixture on Al foil with a small amount of NMP. We used lithium foil as counter electrode and 1 mol·dm^−3^ LiPF_6_ in EC/DMC (ethylene carbonate/dimethyl carbonate; 2:1 *v*/*v*, Kishida Chemical Co., Ltd., Osaka, Japan) as electrolyte to construct a two electrode cell (Hohsen Co., Osaka, Japan). We extracted lithium ion from the sample at a constant current of 0.01 C to x = 0.12. After the termination of lithium extraction, we immediately took the working electrode out of the cell in an Ar-filled glove box to avoid the local cell reaction between the electrode material and the current collector via the electrolyte [28]. Thereafter we washed the working electrode in EC/DMC (2:1 *v*/*v*, Kishida Chemical Corp., Ltd.) and DMC (Kishida Chemical Corp., Ltd.) and then dried in Ar atmosphere.

### 2.2. X-ray Diffraction and the Rietveld Analysis

The sample was set in a sealed holder (Rigaku Corp., Ltd., Tokyo, Japan) with beryllium window in an argon-filled glove box and mounted on a XRD diffractometer (Ultima-lV, Rigaku Corp., Ltd.). XRD experiments were carried out from 15° to 75° in 2*θ* at a rate of 2°·min^−1^ with 0.04° step width by using CuKα radiation. The X-ray tube voltage and current were set to 40 kV and 40 mA, respectively. The six continuously collected XRD data sets were merged to improve the S/N ratio for the Rietveld refinement.

The XRD profiles were analyzed by the Rietveld method using RIEVEC code [29]. The refinements were made assuming the two phases were co-existing, both of which belong to $R\bar{3}m$ symmetry. For the structure refinement, nickel ion was placed at the 3*b* site and oxide ion at the 6*c* site in the hexagonal axis, and the contribution of lithium was ignored. The mole fraction of each phase was calculated from the scale factors.

## 3. Results and Discussion

**Figure 1:**
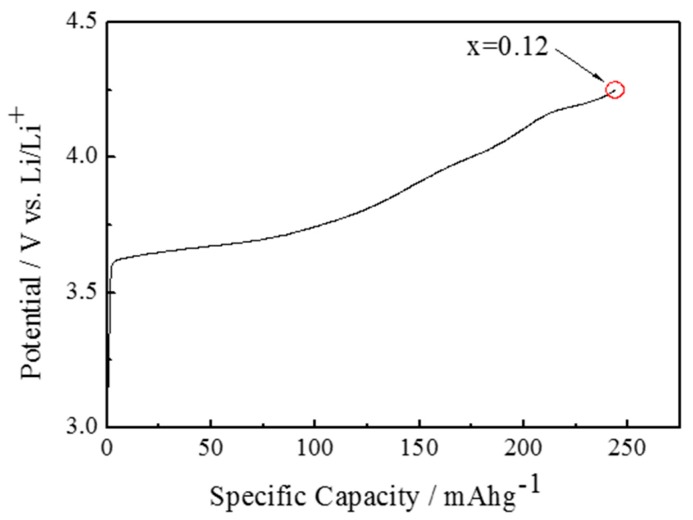
Charge curves of Li_x_Ni_0.874_Co_0.090_Al_0.036_O_2_ cathode material at a constant current density of 0.01 C.

**Figure 2:**
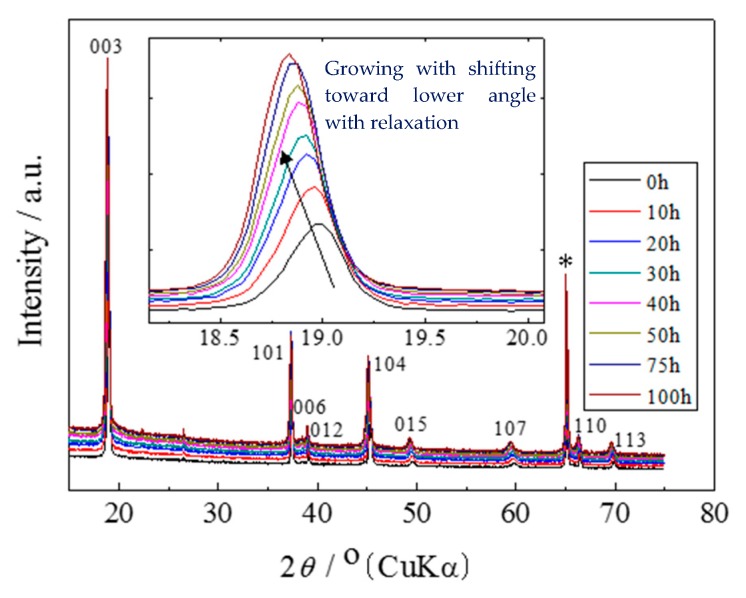
X-ray diffraction patterns of Li_0.12_Ni_0.874_Co_0.090_Al_0.036_O_2_ obtained during relaxation time after the termination of lithium extraction. Diffraction patterns measured after relaxation up to 100 h are superimposed on each plot. Asterisk marks (*) indicate the diffraction peaks of aluminum foil current collector. Diffraction profiles around 003 reflections are enlarged in the inset.

**Figure 3:**
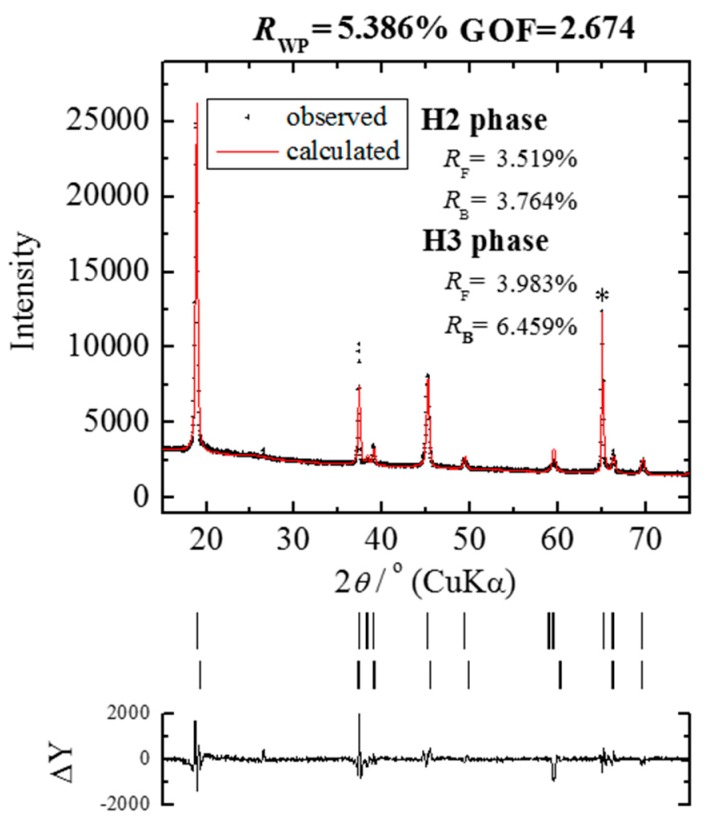
Observed and the Rietveld refined diffraction patterns of Li_x_Ni_0.874_Co_0.090_Al_0.036_O_2_ (x = 0.09) after 30 h of relaxation from the termination of lithium extraction. The vertical lines in the middle section show the positions of peaks calculated for Bragg reflection. The trace (∆*Y*) in the bottom section represents the difference between observed and calculated patterns. The asterisk mark (*) indicates diffraction peaks of the aluminum foil collector.

**Figure 4:**
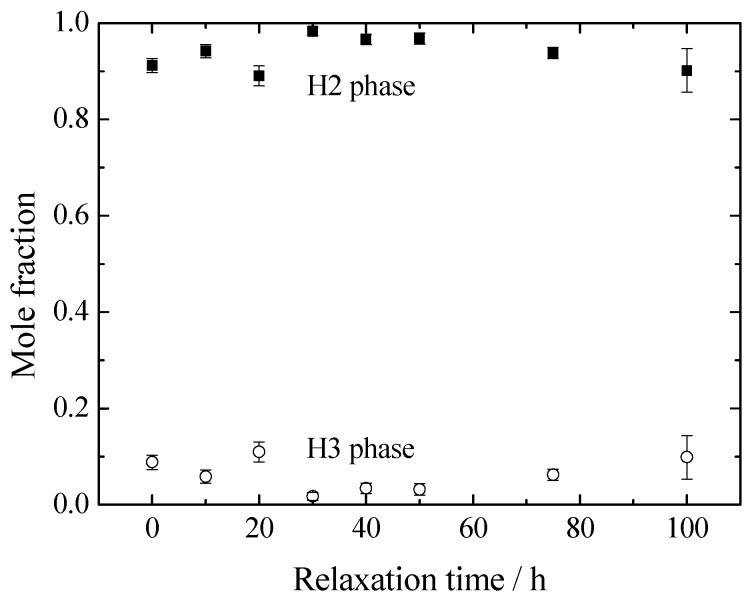
Obtained mole fraction change of Li_0.12_Ni_0.874_Co_0.090_Al_0.036_O_2_ during the relaxation time. Closed and open symbols correspond to the H2 and H3 phase, respectively.

**Figure 5:**
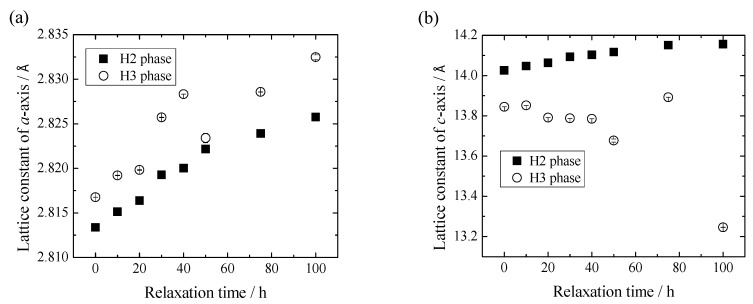
Change in lattice parameters of the (**a**) *a*-axis and (**b**) *c*-axis of Li_0.12_Ni_0.874_Co_0.090_Al_0.036_O_2_ with relaxation time. Closed and open symbols correspond to the H2 and H3 phase, respectively.

For the preparation of the electrochemically lithium-extracted sample, the cell was charged to achieve the amount of lithium as x = 0.12 of Li_x_Ni_0.874_Co_0.090_Al_0.036_O_2_ at a constant current density of 0.01 C. The charge curve is represented in Figure 1, where a small gradient is observed at x = 0.12. X-ray diffraction patterns for Li_0.12_Ni_0.874_Co_0.090_Al_0.036_O_2_ after the termination of lithium extraction are shown in Figure 2. The 003 peaks are enlarged in the inset. They shift toward the lower 2*θ* direction with the relaxation time. The measured and the Rietveld-fitted diffraction profiles with 30 h of relaxation, as exemplified in Figure 3, provide fairly good agreement, yielding a sufficiently low *R*_wp_ value for the following discussion. Some structural parameters after refinement are listed in Table 1 for the initial, 50 and 100 h of relaxed samples. Figure 4 shows the calculated mole fractions of H2 and H3 phases. It can be seen that the H2 phase is predominant and the mole fraction of H3 is approximately 0.1. It was also observed that the mole fractions seem not to vary largely during relaxation. Figure 5 shows the relaxation time dependence of the lattice parameters of the *a*- and *c*-axes. The *a*-lengths are gradually increased with relaxation time for both H2 and H3 phases, while that of H2 is a little smaller. Nevertheless, these variations are still restricted only within 0.01 angstrom. On the other hand, *c*-lengths of H2 and H3 phases increase and decrease respectively with relaxation time, to show a difference of 1 angstrom. Assuming that *c*-length corresponds to the content of lithium ions, Li^+^ migrate from H3 to H2 phases keeping molar ratio constant. Namely, during the lithium extraction, H3 phase involves excess lithium ions in comparison with the equilibrium state.

**Figure 6:**
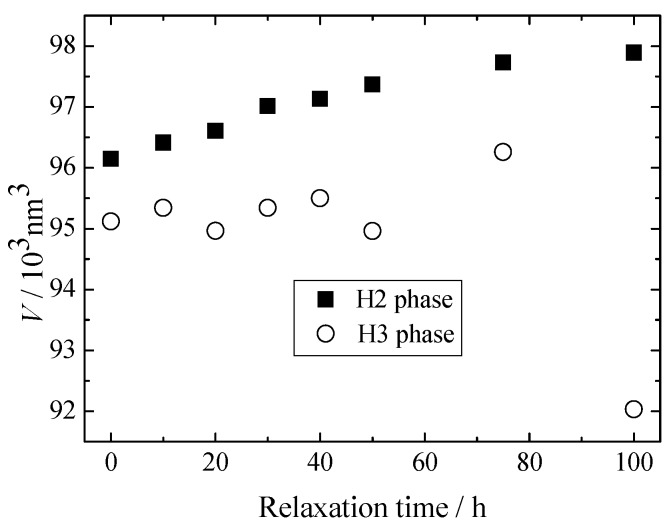
Changes in unit cell volume of Li_0.12_Ni_0.874_Co_0.090_Al_0.036_O_2_ with relaxation time. Closed and open symbols indicate the H2 and H3 phase, respectively.

**Figure 7:**
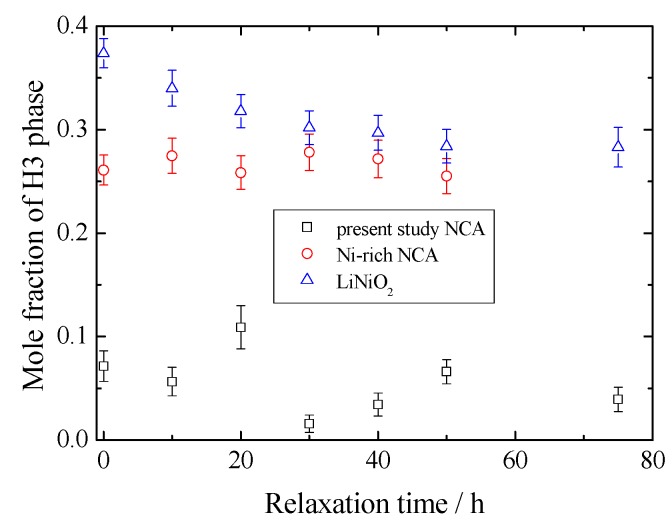
Calculated mole fraction change of H3 phase for LiNiO_2_, Ni-rich NCA and present study NCA.  ∆: LiNiO_2_, О: Ni-rich NCA, □: present study NCA.

Therefore, it is thought that H3 forms from the H2 phase containing still larger lithium ions during charging, after that at the relaxation process lithium migration occurs to achieve a stable condition. Figure 6 shows changes in volume of the unit cell with the relaxation time. The volumes of H2 and H3 phases show the same trend as *c*-length. Figure 7 compares the mole fractions of H3 phases for LiNiO_2_, Ni-rich NCA and present NCA. It can be seen that mole fraction of the H3 phase reduces with the cobalt content, i.e., a larger cobalt introduction stabilizes the H2 phase.

It is also observed that variation of mole fraction during the relaxation time reduces with the content of cobalt. This suggests that since cobalt introduction restricts the unfavorable H2–H3 transition not only in the charging process, but also in relaxation time, NCA improves the cycle performance at highly charged state. In the previous work, we proved that very small amounts of cobalt substitution as well as aluminum largely contribute to the H2–H3 phase transition [18]. Nevertheless, it has been known that transition still occurs in the highly de-lithiated region. Thus, in the present study, increasing the cobalt concentration, H2–H3 transition and relaxation behavior has been observed. We are now investigating the relaxation of Co-rich NCA after the electrochemical lithium extraction down to x < 0.12 to compare with these materials. We are planning to discuss the structural variability of NCA series after the data collection of the above system.

## 4. Conclusions

We have performed a relaxation analysis on Li_x_Ni_0.874_Co_0.090_Al_0.036_O_2_ to study the structural variability at the deeply Li extracted region for x = 0.12 and compared with previous LiNiO_2_ and Ni-rich LiNi_0.933_Co_0.031_Al_0.036_O_2_ systems concerning the cell performance of NCAs. It was found that during the relaxation time, the *c*-length of the H2 phase increases while that of H3 phase decreases, keeping with the molar ratio. The H3 phase, with larger lithium ions at the initial stage, provides the lithium ions to the H2 phase during the relaxation time. When comparing with the previously reported LiNiO_2_ and Ni-rich NCAs, it is found that higher content of Co restricts the unfavorable H2–H3 transition, which would attribute to cell performance.

## Figures and Tables

**Table 1:** Refined structure parameters of Li_0.12_Ni_0.874_Co_0.090_Al_0.036_O_2_ for initial state, and after 50 and 100 h of relaxation.

Relaxation Time	Mole Fraction		*c*/Å		Oxide Ion Coordinates		*R*_wp_
	H2 Phase	H3 Phase	H2 Phase	H3 Phase	H2 Phase	H3 Phase	
0 h	0.91 (1)	0.09 (1)	14.062 (2)	13.843 (9)	0.2316 (7)	0.268 (4)	5.09
50 h	0.97 (1)	0.03 (1)	14.116 (1)	13.755 (8)	0.2341 (8)	0.218 (10)	5.71
100 h	0.90 (4)	0.10 (4)	14.156 (10)	13.246 (8)	0.2329 (7)	0.298 (9)	5.40
